# Commentary for Special Issue on Advancing Health Equity Among Black Communities: Implications for Research Funders (and Researchers)

**DOI:** 10.1007/s11121-023-01636-9

**Published:** 2024-01-08

**Authors:** Kimberly DuMont

**Affiliations:** William T. Grant Foundation, New York, NY USA

Prevention science pledges to promote individual and community health and well-being. It does this by (a) scientifically investigating issues that threaten or undermine health, (b) studying policies, programs, and practices to prevent and reduce harm from these issues, and (c) examining strategies about how to use knowledge from research, data, professional expertise, and other forms of evidence. The collection of articles in this special issue offer theory and empirical evidence about how to improve on these efforts and better advance health equity among Black communities.

I write this piece from the perspective of a private research funder at the William T. Grant Foundation. Like other public and private funders, we play a role in the research ecosystem (Boyd et al., 2023). Funders provide needed resources for prevention science to deliver on its commitment. The authors of the articles within this special issue provide both public and private funders with ideas and actions that will more urgently and responsively advance health equity, although what ideas and actions a funder can prioritize depends on the resources and constrains of their funding institution. As Murry and colleagues (2022) encourage, we need to re-envision and retool the research ecosystem to rebuild prevention science.

*We need to critically examine when and how current theories, measurement tools, methodologies, and the design, development, and testing of prevention interventions may perpetuate injustice, and how they can be radically re-envisioned, retooled, and rebuilt to dismantle racism and promote equitable health for minoritized communities (*Murry et al., 2022*, p. 2).*

The editors of this special issue and the authors of the articles here and elsewhere (Boyd et al., 2023; Fletcher et al., 2021; Hassan et al., 2021; Murry et al., 2022; NIH Advisory Committee, 2021; PEAK Grantmaking, 2020; Ray et al., 2023) invite researchers, research funders, advocates, communities, and policymakers to revisit funding structures and priorities. They urge us to fund research that more directly addresses structural and individual racism. The current collection is exceptional in that it offers both a critique of where the research field has been and concrete strategies and ideas for promoting health equity in the future.

What follows are my reflections, as a private funder of research, on how we and other funders might take up this invitation to change a long, repeating, and unhealthy narrative, so that funders, researchers, and advocates can do better by and with Black Communities. I encourage us to engage with at least three of the authors' ideas and requested actions. First, question how we frame calls for proposals and prioritize focus areas and research questions. Second, privilege a broader array of research methods and measures than is current practice so that we can respond to the full range of research questions being asked. Third, expand the expertise that we include in the review of proposals and allocation of awards.

## Question the Frames that Guide Calls for Proposals and Prioritization of Research Questions

Funders’ requests for proposals frame how we understand and respond to health problems and what questions researchers prioritize. For example, each study in this issue makes clear that racism has been and continues to be a source of stress for Black communities, but despite the clearly documented consequences of this structural factor (Barbarin et al., 2022; Summers-Gabr, et al., 2023), most interventions have focused on individuals and families (Boyd et al., 2023; Ray et al., 2023); a smaller number have focused on the access and quality of supportive systems (Roman, 2022); and a rare few have studied structural factors or racism itself (Hassen et al., 2021; NIH Advisory Committee, 2021). This is due in part to the prevailing frame that guides research funders’ investments in prevention science, which focuses on individuals, specifically on supporting and enhancing the skills of those targeted by racism and discrimination. The authors of the articles in this special issue urge us instead to support research on and using anti-racist strategies, including studies that pursue more culturally relevant and strength based-approaches (Barbarin et al., 2022; Berkel et al., 2022; Murry et al., 2022; Temple & Varshney, 2023), and which engage those most directly affected in the design of the research (Woods-Jaeger et al., 2022). But this call for change comes with guidance about ethical approaches (Woods-Jaeger et al., 2022) and cautions about positionality (Wooten, 2023). Similarly, the authors recommend approaches that focus on the actors and environments that can perpetuate racism (Temple & Varshney, 2023). The aspiration is for new paradigms, methods, and measures that usher in health equity.

One takeaway for funders is to focus on the assets and values of Black people (Fullwood, 2011) and to shift the lens away from deficit models. This shift may help to reduce the stigma that the research enterprise contributes to how Black communities are seen (Christophe et al., 2022; Hatzenbuehler, 2016; Pattillo, 2021). A more asset-based lens is evident in several articles in this special issue, although disparities and challenges to health and mental health still anchor the research. For example, Berkel et al. (2022); Roman (2022); and Dinizulu and colleagues (in press) present findings from evaluations of interventions that reduce the consequences of stress from racism and discrimination by bolstering individual and family skills and practices. They argue for and pursue this research through a cultural lens and leverage assets in individuals and families while also responding to needs. A 2021 essay by Mary Pattillo proposes a framework that moves even farther from a deficit framing. In “Black Advantage Vision: Flipping the Script on Racial Inequality Research,” Pattillo (2021) invites research that elevates “the strengths, resilience, care, and accomplishments” (p. 5) of Black people, as well as the study of areas where Black people outperform White people. We see steps in this direction among those interventions and studies designed to amplify individuals and families’ strengths (e.g., racial socialization, Black pride, and agency).

Funders can also leverage calls for proposals to emphasize system reform and improvements. Although not featured in this special issue, there is a promising and growing body of research on how the removal and redesign of administrative burdens and systems supporting child care (Barnes & Gennetian, 2021), mental health (Rodgers et al., 2022), health (Pérez-Stable & Rodriquez, 2023), child welfare (Barnes & Petry, 2021), and economic services (Ideas42, 2022) can open the door toward engagement with supports, reduce system bias, and contribute to improved emotional, physical, and economic health.

As urged by the authors of the articles in this special issue, funders are also invited to thoughtfully call for the study, design, and evaluation of more structural approaches (Boyd et al., 2023; Berkel et al., 2022; Murry et al., 2022) to advance health equity in Black communities and combat the lack of robustness of interventions when implemented in discriminatory and stigmatizing climates (Hatzenbuehler, 2016). Findings from several studies underscore the value of designing for viability within a geographical or political context, and yet these same studies reveal the limitations of mitigating harm while structural and more systemic factors persist (Hatzenbuehler, 2016; Summers-Gabr et al., 2023). A promising path forward is funding the development and study of culturally anchored interventions and interventions that directly equip participants to navigate racism and its harms, which are more robust (Anderson et al., 2023).

More directly, funders can incentivize theory and empirical study of change strategies that address the pernicious, pervasive nature of racism to foster broadscale and upstream societal change (McCambly & Colyvas, 2022; Murry et al., 2022; Ray et al., 2023). This includes support for qualitative work to extend, remodel, or refute existing theoretical frameworks as well as mixed methods design to examine whether there is support for newly proposed change strategies. The call to directly address and prevent racism also reinforces conclusions of the NIH Advisory Committee (2021) which called for research to understand systemic racism in research studies and in the scientific workforce.

The authors of the articles in this volume offer ideas for jump-starting funding for research in these areas. They encourage interventions and research questions that more directly align with the identified systemic contributor and tackle racism and discrimination head on. Murry and colleagues (2022), for instance, call for the design and evaluation of interventions that target institutional racism in training institutions and public systems. The funding organization that I work for, the William T. Grant Foundation, is assembling a growing portfolio of studies on interventions that aim to disrupt interpersonal and cultures of racism by working with White students, families, educators, and system leaders who contribute to unhealthy systems, and through proximity or position can disrupt processes and structures that perpetuate harm. Hurd et al. (2022), for example, studied strategies to encourage White bystanders to confront online racial discrimination directed toward Black college students attending a White institution. Study findings suggest that White students are motivated when they are aware the posts cause harm to their Black perceives and are equipped with guidance about how to engage (Hurd et al., 2022). Hurd and colleagues also note perceived social norms also matter, and White students are more likely to confront a post when confronting discrimination is a perceived norm. (Hurd et al., 2022). Importantly, Black students reported that harm caused by exposure to online racial discrimination was lessened when the discrimination was challenged by their White peers. As the articles here repeatedly demonstrate, racism is baked into structures, culture, and interpersonal exchanges, and everyone has a role to play in dismantling it. Funders can advance visions for dismantling racism while also funding research that elevates and advances the assets and advantages that are present and plentiful in Black communities.

## Privilege a Broad Array of Methods to Respond to the Full Array of Research Questions

The methods and measures used in the research—the *how* of research—are as important as *what* is researched. Calls for proposals directly or indirectly privilege some methods and measures and discourage or exclude others. Boyd and colleagues (2023) argue that research methods should be designed to respond to a study’s research questions and aims. Funders calls for proposals can privilege studies with research questions and methods that have historically not been funded and studies with questions that are frequently funded but involve different methods that can reveal new insights. Murry and colleagues (2022) invite funders and researchers to revisit how the methods and measurement tools used perpetuate or help dismantle racism. Two responses might include: (1) broadening the range of methods privileged in our calls for proposals from what is currently is invited and (2) investing in new measures that allow for research pursuits related to systemic and structural change, including measures that reflect more complete and culturally anchored indicators of well-being and health.

### Broadening the Range of Methods

Methods are essential to the research enterprise. They facilitate the generation and analysis of data to address research questions. Yet there is considerable diversity in the questions that are asked, and funders need to support a full range of methods and ways of knowing. Fletcher et al. (2021) argue that addressing the structural roots of racism, mitigating its harms, and contributing to better public health systems requires the ethical engagement of those communities that have suffered the greatest inequities. This theme is present in the current special issue and elsewhere (e.g., Skelton-Wilson et al., 2021). Woods-Jaeger et al. (2022) present a detailed account of what ethical engagement of youth in research might look like. Woods-Jaeger et al. (2022) developed a community-based prevention strategy that actively resists structural racism, Youth Empowered Advocating for Health (YEAH). This article is notable for the authors’ transparency in describing the work, as well as their aims to address structural racism with a team of varied experts, including lived and local expertise, and to strive towards the promotion of a positive outcome—equitable and sustainable health for and with Black communities. Community and researcher contributions were made throughout the project lifeline, from inception, to design, to implementation and evaluation, to sense-making.

For broader scale recognition of this approach, funders must also take notice of the rewards and resources required when engaging a more comprehensive set of experts. This means adequately compensating a comprehensive set of team members for the varied types of expertise that contribute to and guide the research (Mihalec-Adkins et al., 2023; Powers & Tiffany, 2006). Funding a more complete team structure also means investing more heavily in the team’s infrastructure, with staff time dedicated to organizing multiple team members and nurturing a varied set of relationships throughout the life of the project. In addition, there may be a need for a funded initial period where agreements, agendas, and processes for working together evolve to cement roles and relationships. In turn, this may mean that project budgets will increase. Yet, as illustrated in the articles, and as my organization is experiencing in our Institutional Challenge Grant program, supporting partnerships and teams that comprise different roles, expertise, organizations, and community locations often benefits the usefulness and use of the resulting research (Gamoran, 2018; Tseng & Nutley, 2014).

#### Investing in New Measures

Measures help research teams operationalize their constructs in ways that allow for descriptions of the construct at a moment in time. As the focus of funders and researchers shift from individuals and systems to structures and cultures, from limited to expanding ideas about racism and promotive mechanisms, from dysfunction and unhealthy outcomes to well-being and healthy outcomes, and from gaps to equity, new measures will be needed to describe these indicators and phenomena and be sensitive to change (Boyd et al., 2023). Funders have an important role in paving and resourcing avenues for the development and validation of measures that allow for the characterization of new constructs, mechanisms, and outcomes that attend to variation and allow for monitoring of change over time or as a result of intervention.

Boyd et al. (2023) provide a rich and nuanced description of some of the opportunities, challenges, and considerations as this work moves forward, and they do so in a way that is specific to health equity. At the William T. Grant Foundation, and relevant to this discussion, we recently funded Husain Lateef to develop and validate a measure of Afrocentric Cultural Socialization. Based on earlier work (Husain, 2023), Lateef argued this measure was necessary to better understand the process by which Black parents, caregivers, and mentors share beliefs, cultural norms and traditions, and pride to Black children to bolster educational success and health. Lateef and colleagues are also focused on the intersecting identities of race and gender, with a particular focus on Black males. Additionally, the team will conduct a scoping review of existing Afrocentric measures, convene an expert panel to provide guidance on measurement development, and hold cognitive interviews with adolescents, while also conducting analyses with items from existing measures to assess criterion validity, convergent validity, and divergent validity. This type of measurement work is needed to reveal understudied mechanisms and elevate positive health outcomes. Funders have an opportunity to invest in the time and resources it requires to develop other valid and reliable measures that allow studies of health equity to advance.

## Expand the Expertise that is Included in the Review of Proposals and Allocation of Awards

Just as important as *what* is being researched and *how* research is conducted, is *who* participates in the grantmaking process to determines what ideas are elevated, how they are pursued, how they are evaluated, and who receives resources. In this way, funders play a role both in promoting equity and exacerbating inequities. Funders’ decisions about research topics (Hoppe et al., 2019; Lauer et al., 2021) and review processes (Ginther et al., 2011; NIH Advisory Committee, 2021) are consequential for funding opportunities and research awards. And while prior funding for prevention research has contributed to important programmatic work, better social and economic policies, and tools for practice that improve conditions for some, the articles in this special issue focus attention on the inequities that challenge the health of Black community members.

Funders have an opportunity to increase investments in the resources and vision within Black communities (Pattillo, 2021) that could help embed structures, processes, and practices in our society and systems that promote health. To date, many research funders have excluded those who sit outside the research community in their grantmaking beyond being study participants. Yet there are cooperative, mutual aid, and movement models of funding that demonstrate different ways of operating than what is generally practiced in public and private research funding (Fullwood, 2011). While a re-imagining of funding structures will take time, we can immediately respond to calls to include the voices of those most often affected by the ideas and recommendations pursued in research. Research has repeatedly demonstrated the value of meaningfully engaging community members to deepen understanding of lived experiences (Chicago Beyond, 2019; Smith & Smith, 2005), to inform the design and viability of prevention and interventions efforts (Debnam & Kumodzi, 2021; Ishimaru & Takahashi, 2017), and to ground and expand the interpretation of findings and product development and dissemination efforts (Chicago Beyond, 2019; Powers & Tiffany, 2006; Woods-Jaeger et. al., 2022). By extension, there is also value in revisiting who drives public and philanthropic funding agendas, who is invited to review and evaluate proposals, and who is engaged in the decision making that influences resource allocation.

Funders have an opportunity to incentivize and support the sustained inclusion of Black communities to better anchor research in lived experiences and elevate insights that strengthen or reorient research to enhance the contributions of prevention science. In addition, more diverse funding teams will help to protect against bias that creeps in through our networks, attraction to the familiar, conforming, and confirming (PEAK Grantmaking, 2020). There is space for funders to move beyond consultation and light touch advisory models to routinely and meaningfully engage a broader set of contributors to their grantmaking processes. Here, we might learn from the examples below, and others:

- PCORI (Patient-Centered Outcomes Research Institute), to engage patients in all aspects of their research grantmaking
- The Annie E. Casey Foundation’s Juvenile Justice Youth Advisory Council, to inform its programmatic work in this area
- The Ford Foundation, to formalize a mechanism for ongoing input from leaders and members of the disability community
- The Mayoral Office of Baltimore, to initiate a youth council that informs its policies and investments related to youth

It is time for thoughtful forward movement that centers Black communities to devise ethical and beneficial ways to engage community members in grantmaking. Black community members representing different roles and experiences can elevate, add to, and discourage the importance of a study topic, anticipate feasibility concerns that researchers have missed, introduce new items for measures and add nuance about a study’s context, and add nuance to the understanding of Black community members experiences. Importantly, Black community members can also help reframe narratives, inform research questions, instruct data collection, provide insights to help with sense-making and research use, and help anticipate and understand unwanted consequences. Centering Black communities would result in a more complete set of reviews for research teams, be theoretically generative, and increase funders’ awareness of our blind spots and potential harms. These assessments would inform what ideas receive funding. In turn, participation in the design and implementation of grantmaking may allow for greater agency in research that promotes health equity.

These changes will require sustained engagement, education, and training among funders’ staff and leaders, as well as commitment of time and dollars. Additional and intentionally designed infrastructure will be required to facilitate expansions and shifts in who we engage and how we structure and practice our grantmaking. Such infrastructure is essential for navigating community differences, demands of the work, power dynamics, and change. In sum, to better achieve health equity in Black communities, we need higher quality grantmaking that embraces a more diverse set of voices to inform the research we fund.

## Closing Call to Action

I hope this volume lives up to editors Katrina Debnam, Caryn Rodgers, and Paula Smith’s aspirations to build a research ecosystem that advances health equity for Black communities. Funders have important role to play in fulfilling this aim; we are part of the prevention science ecosystem. Funders are needed to usher in new, anti-racist approaches to funding and research. To do this well, we must embrace input from researchers, advocates, and communities at every phase of the research so that funders, researchers, and communities more readily know and understand when approaches are on solid ground, and how our grantmaking, review, and post-award policies and processes maintain the status quo and cause harm. If we do not allow critiques of our methods as funders, funders are unlikely to change in ways that move researchers and others toward the aspirations outlined. We share the goals to contribute to qualitatively better health infrastructures, access, experiences, and outcomes for the individuals that compose the Black communities the authors recognize and celebrate.

Change is never easy, but if we can fund research that identifies and informs ways to disrupt the structures and mechanisms that perpetuate harm and contribute to better systems and life experiences, we will move the needle on health equity. The ideas discussed above are not exhaustive but do showcase some immediate and meaningful steps we might take as a community to re-envision how we structure our calls for proposals, what research questions and methods we support, and who is engaged to make those decisions. We will surely need to do more to reach the editors’ aspirations, but this should not prevent us from acting now. Further, we must also diversify our support to improve how what is learned from research is used in ways that promote health equity.

Research does not speak for itself (Tseng & Nutley, 2014). It requires interpretation (Doucet, 2021). It requires advocates to broker understanding and use (Crowley et al., 2021). It requires infrastructure investments so that communities, organizations, and decision makers can integrate research in their routines (DuMont, 2019). And this, too, is funders’ investment to support.
